# Practical Implementation of the Comprehensive Geriatric Assessment to Optimise Care for Older Adults with Cancer

**DOI:** 10.3390/geriatrics8010018

**Published:** 2023-01-23

**Authors:** Laura Tack, Patricia Schofield, Tom Boterberg, Rebecca Chandler, Christopher N. Parris, Philip R. Debruyne

**Affiliations:** 1 Department of Medical Oncology, Kortrijk Cancer Centre, General Hospital Groeninge, 8500 Kortrijk, Belgium; 2 Department of Human Structure and Repair, Ghent University, 9000 Ghent, Belgium; 3 School of Nursing and Midwifery, Faculty of Health, University of Plymouth, Plymouth PL4 8AA, UK; 4 School of Allied Health, Faculty of Health, Education, Medicine and Social Care, Chelmsford CM1 1SQ, UK; 5 Medical Technology Research Centre (MTRC), School of Life Sciences, Faculty of Science and Engineering, Anglia Ruskin University, Cambridge CB1 1PT, UK

## 1. Introduction

Whilst cancer remains a very serious health problem at any stage, cancer combined with increasing age creates an even more challenging situation for health care providers. Age is the principal non-modifiable risk factor for all cancer types. There is a rising incidence of cancer among older adults worldwide. In Belgium, the highest incidence rate is situated in the 80–84 age group [1]. In 2020, 60% of women and 70% of men in Belgium were aged 65 years or older when diagnosed, an increase of 6% for both sexes compared to 2010 [1]. Older adults with cancer can be a vulnerable group of individuals, as they present with unique characteristics that have naturally developed as a consequence of aging, such as altered physiology, functional impairment, and comorbidities [2].

In geriatric oncology, there is an awareness of the heterogeneity of the population of older adults with cancer and the spectrum of impairments that may be experienced. Decision making should be based on the patient’s functional age rather than the chronological age, as chronological age alone is often a poor indicator of the physiological and functional status of older adults [3]. Some older adults will present as independent individuals, whereas others may be at a moderate risk of health deterioration or even have a high risk of functional decline or mortality. These different profiles, ranging from fit over vulnerable to frail, can be determined through a geriatric assessment, and more extensively through a comprehensive geriatric assessment (CGA). The CGA is the gold standard in geriatric oncology to identify patients at a high risk of adverse outcomes and optimize cancer and overall management [4,5]. This multidisciplinary, in-depth evaluation helps physicians to assess the objective health of the older adult while evaluating vulnerabilities in different age-related domains such as functional status, physical performance, cognition, nutrition, social status, emotional status, comorbidity, and polypharmacy. The appropriate screening and identification of vulnerable or frail older adults is an important step to administer a safe and effective cancer treatment. Evidence-based data based on the geriatric profile are essential to determine potential risks and benefits older adults with cancer could encounter during and after cancer treatment. The use of geriatric screening tools such as the CGA helps physicians to develop a coordinated plan for cancer treatment and to guide interventions tailored to the individual. The integration of general geriatric principles focusing on the patient rather than the tumour represents the first step towards individualized treatment decisions, and consequently, the avoidance of over- and undertreating older adults with cancer.

In a recent report on Clinical Cancer Advances, the American Society of Clinical Oncology (ASCO) stressed the importance of an equal opportunity to participate in, be recognized for, and benefit from research for all populations [6]. Amongst others, the optimisation of care for older adults with cancer was mentioned as one of the research priorities in order to accelerate progress against cancer, with the following focus areas: (1) the use of practice-based data to better understand the efficacy and toxicities of cancer treatments; (2) the development of standardized methods to characterize physiologic aging, such as geriatric assessment; and (3) testing the role of geriatric assessment-guided management in improving outcomes using personalized care, in order to minimise the undertreatment of fit patients and the overtreatment of vulnerable or frail patients.

## 2. The CGA as a Tool to Assist in the Management of Cancer-Related Toxicity

The first challenge highlighted by the ASCO is the use of practice-based data to better understand the efficacy and toxicities of cancer treatments in older adults with cancer. Indeed, the underrepresentation of (vulnerable) older adults with cancer in phase II/III clinical trials continues to pose a major problem. This forces physicians to rely on their own clinical expertise, rather than the extrapolation of evidence-based data to assess the benefits and risks of a particular therapy for an (vulnerable) older adult with cancer. The outcome of a case-control study by our research group supports that the majority of older adults with cancer included in clinical trials have a fit profile (4). Those with a vulnerable profile, who represent the majority of older adults with cancer in real practice, are underrepresented in clinical trials [7].

This calls for research to perform subpopulation studies or to include older adults with cancer in later stages of clinical trials. Another tool to address this challenge is the use of artificial intelligence (AI). Platforms using AI and natural language processing represent a potential tool to obtain valuable insights about the dynamics of patients’ reserves and cancer treatments in a plethora of cancer types, generating a snapshot of the real-world patient population.

At present, our research group is preparing a research project on the effectiveness and toxicity of combination therapies for older adults coping with stage IV kidney cancer. In this post-marketing observational study, a trial-specific sample size calculation will be applied to recruit separate cohorts of fit and vulnerable older adults with cancer for each immunotherapy combination regimen. Fleming’s Two-Stage Design with an admissible adaptive design will be utilised as a formal stopping rule for a high rate of adverse events leading to trial discontinuation (as compared to the data provided in the respective landmark trials). This could lead to the avoidance of regimens that are associated with too much toxicity in vulnerable (as compared to fit) older adults with stage IV kidney cancer (in collaboration with Dr S. Kim [8]). The serial performance of a geriatric assessment will help us to identify which older patients will truly benefit from immunotherapy combinations and at what risk (discerning fit versus vulnerable older patients). With this project, we aim to contribute to the body of knowledge on how to appropriately treat this patient population, responding to the ASCO’s call for achieving equity in cancer research and hereby representing all the different individuals.

The use of AI to provide practice-based data to better understand the efficacy and toxicities of cancer treatments represents another tool to assess excess toxicity and the maintenance of beneficence in real life populations. In that regard, the general hospital Groeninge collaborates on a multicentric project of LynxCare^®^ (Leuven, Belgium) on the Real-World Evidence Observational Study of Cancer Patients Treated with Immune Checkpoint Inhibitors [9]. These tools and their outcomes may contribute to greater equity in cancer research and, therefore, represent all the different individuals that are being treated. For our research group, these methods of AI will also be exploited to determine the outcomes of the geriatric assessment and the according management contributing to the optimisation of the CGA as such.

## 3. CGA in Routine Practice: The Kortrijk Older Adult Oncology Care Model

The second challenge set by the ASCO refers to the development of standardized methods to characterize physiologic aging. Indeed, a patient’s chronological age is often a poor indicator of the physiological and functional status of older adults [3]. There is, thus, a need for a more reliable tool to assess one’s objective health and to perform clinically justified decision making regarding the treatment plan of an older adult with cancer.

Potential solutions include the identification of biomarkers of aging and clinical pharmacology in older adults. A widely used tool already available for the characterization of physiologic aging is the use of a geriatric assessment such as the CGA. Although the importance of the CGA is promoted by international organisations, including the National Comprehensive Cancer Network (NCCN), its implementation may lag behind, as it involves a dedicated team accomplishing the geriatric assessments, which is embedded as routine practice in clinical settings.

**Figure 1:**
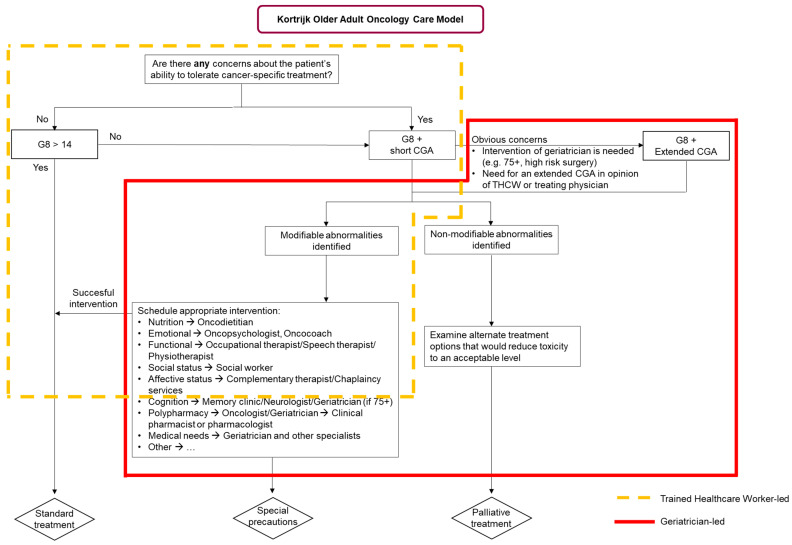
The Kortrijk Older Adult Oncology Care Model.

With the support of the National Cancer Plan in Belgium, the “oncogeriatric clinic” was founded at the OECI-designated Kortrijk Cancer Centre in 2010. This clinic is staffed by both scientific research associates responsible for the research aspect and oncopsychologists responsible for daily practice, hereafter referred to as trained healthcare workers (THCWs). Throughout the years, we have optimized the workflow of the oncogeriatric team at the Kortrijk Cancer Centre, which resulted in the “Kortrijk older adult oncology care model” (Figure 1). The initial step in routine practice is the administration of the Geriatric 8 (G-8) questionnaire. The G-8 screening tool identifies those older adults with cancer who would benefit from a CGA [10,11], and indicates quality-adjusted survival [12]. This two-step approach is performed by the team of oncopsychologists of the oncogeriatric clinic who are trained to administer the geriatric assessments (THCW-led pathway). If there are no concerns about the patient’s ability to tolerate cancer-specific treatment, the patient will be screened with the G-8 screening tool and sequentially receive standard cancer treatment when G-8 negative. However, if there are concerns and the patient effectively scores 14 or less on the G-8, the patient undergoes a short CGA (sCGA) performed by the THCW (Table 1). In the case of obvious concerns, such as a high-risk surgery, older adults with cancer can be referred to the geriatrician-led pathway (Figure 1). The geriatrician-led pathway involves an in-depth evaluation of multiple domains of the CGA, including extended CGA (eCGA), at the geriatric day clinic by a multidisciplinary team, which is supervised by the geriatrician. This is often combined with scheduled pre-operative or staging examinations at the geriatric day clinic to mitigate time toxicity for the patients.

## 4. Testing the Role of Geriatric Assessment-Guided Management

To continue the challenge of the development of standardized methods, this focus area goes hand in hand with the according management of the defined vulnerabilities. The integration of the CGA in oncology has provided several advantages, promoting its use in routine cancer care. They include, but are not limited to, guidance in treatment decision, the prediction of chemotherapy toxicity, and the prediction and improvement of survival and quality of life [12,21,22]. Nevertheless, it should be noted that conducting a CGA alone does not add any value to the patient’s treatment plan without informing consequent tailored interventions. The latter was also stressed in the third focus area by the ASCO: it is a challenge to test the role of geriatric assessment-guided management in improving outcomes using personalized care, in order to minimise the undertreatment of fit patients and the overtreatment of vulnerable or frail patients. Besides toxicity management, the CGA is also essential to meet the quality standard stipulated by the International Psycho-Oncology Society (IPOS), stressing the importance of the integration of a psychosocial domain into routine care to provide quality cancer care [23].

According to the ASCO, it remains a challenge to provide supportive care interventions and care delivery interventions to improve outcomes using personalized care. Potential solutions entail a decent workflow on specific vulnerabilities uncovered by performing a CGA, in order to provide each older adult with cancer with the cancer care that is required without overshooting the vulnerabilities, but also without undertreating those older adults with a fit profile.

The CGA can adequately identify several vulnerabilities that could require an appropriate referral. For example, a referral to a dietitian may be made in the case of unintentional weight loss in the last 6 months, or offering access to complementary therapies such as Emotional Freedom Techniques if the patient would present with subjective cognitive problems [24]. When standard treatment is not an option for an older adult with cancer, it might be feasible to receive cancer treatment when taking special precautions, such as dose modification or opting for a less toxic regimen. For older adults who appear to have a frail profile or non-modifiable abnormalities, the geriatrician or oncologist will examine if alternate treatment options are available. If not, these patients will receive supportive or palliative care. Further examples of modifiable abnormalities that require the appropriate scheduling of one or more interventions performed by the multidisciplinary geriatric oncology team are mentioned in Figure 1.

Examples to support the importance of the geriatric assessment-guided management may be delivered by our research group via the earlier mentioned example of the cohort study of fit and vulnerable older adults with stage IV kidney cancer, which will be set up for four immunotherapy combination regimens. The performance of serial CGA throughout the treatment period will be used to test the role of geriatric assessment-guided management to minimise the undertreatment of fit patients and the overtreatment of vulnerable or frail patients.

Previously, our research group has performed extensive research on several psychosocial domains included in the CGA [23]. One of the current challenges we address in the population of older adults with cancer is the assessment of depressive symptoms. Depression has been identified in approximately 28% of older adults with cancer and its prevalence can have a significant impact on the patient’s ability to receive life-sustaining treatment for their cancer [25]. Within the CGA, the emotional status is assessed by the 15-item version of the Geriatric Depression Scale (GDS-15). Yet, since the GDS is only a screening tool, the GDS-15 is often considered too time-consuming in clinical practice. Moreover, it contains a number of inappropriate questions that may also lead to an inaccurate assessment of depression in older adults with cancer. This domain thus requires optimisation where the GDS-15 might be replaced by a shorter screening tool. A pilot study by our research group identified the Patient Health Questionnaire two-item version (PHQ-2) as a valid screening tool to accurately assess depressive symptoms in older adults with cancer (manuscript by Tack L et al. in preparation). This pilot study is an example of the continued development of the CGA into a more effective and tailored aspect of geriatric cancer care.

## 5. Implementation of the CGA in Daily Oncology Practice

The integration of the geriatric assessments in routine cancer care would represent a major added value. Several international oncology societies have published guidelines on the different domains included in the CGA and the performance of the CGA by a multidisciplinary geriatric oncology team, including geriatric oncology nurses, dietitians, psychologists, oncologists, geriatricians, and other health care professionals [26,27,28]. However, in practice, given high workloads in healthcare, we doubt the universal recommendation of a full multidisciplinary team to perform the CGA in all patients at every transition (e.g., change in line of treatment). Given the obvious benefits of geriatric cancer care, psychologists and other members of the multidisciplinary oncogeriatric team should receive adequate education to undertake CGAs and to challenge barriers related to culture, language, misinformation, and cancer stigma. 

The increasing population of older adults with cancer comes along with a high socioeconomic burden, which calls for an effective use of the available geriatric assessment tools and healthcare professionals. A Belgian national consortium led by researchers of the KU Leuven identified 70% of the older adults with cancer screened by the G-8 as vulnerable (G-8 ≤ 14) [29]. To limit the use of resources and staff, we apply a two-step approach at the Kortrijk Cancer Centre (Figure 1). In the first step, our oncopsychologists are the THCWs who conduct the G-8 and sCGA and simultaneously introduce the availability of psychological support to the patients. The two-step approach allows the first step to be performed by other healthcare professionals, such as an advanced practice nurse, an occupational therapist, a physical therapist, or a nurse at the geriatric or oncology department, when provided with adequate training to become a THCW. This two-step approach facilitates the implementation of geriatric assessments, increases coverage, and makes it feasible for implementation in routine practice. In the future, tele-sCGA [30] and the validation of shorter screening tools (a research focus of our research group [16,31,32,33]) to be applied in the sCGA could further facilitate implementation in oncology practice.

To continue with the efficiency of performing a CGA, we trust a centred approach with a THCW identifying vulnerabilities as the first step. This does not neglect the importance of a multidisciplinary geriatric oncology team: in our view, they are complementary to the role of the THCW. In the outlined two-step approach, the responsibility for the management of the identified vulnerabilities represents the second step, which should be assigned to this team. Therefore, it is certainly necessary to have a multidisciplinary geriatric oncology team in place to start addressing the outcome of both the sCGA and eCGA, and accordingly schedule the necessary interventions (Figure 1). They also bear the responsibility of adding the principles of geriatrics into the daily practice of geriatric oncology, which requires training specific to their discipline with focus on the older adult with cancer. This last notice was recently highlighted by SIOG’s patient advocate Ms. Beverly Canin, who stated “*research training should include how to engage with patients not just as subjects or peripherally but as collaborators and partners*”.

In conclusion, we propose the implementation of the CGA as a two-step approach performed with older adults who appear vulnerable on the G-8. There is a call from international organisations to standardize cancer care, including the performance of geriatric assessments. We believe there is great potential in the performance of the screening and CGA by a THCW, complemented by a multidisciplinary oncogeriatric team available to manage the identified vulnerabilities. This requires upskilling of the healthcare professionals involved, which in turn will raise awareness and, thus, also improve the quality of cancer services for older adults.

## Figures and Tables

**Table 1:** Overview of the short Comprehensive Geriatric Assessment (CGA) performed by the trained healthcare worker at the Kortrijk Cancer Centre [13].

Domain	Description	Assessment Tool
Functional status	Assessment of limitations in physical function activities Assessment of ability to complete activities required to maintain independence	Activities of Daily Living (ADLs) [14] Instrumental Activities of Daily Living (IADLs) [15]
Physical status	Number of falls in the past year	Not applicable
Social status	Evaluation of living conditions, marital status, education, professional homecare	Not applicable
Cognition	Assessment of patient’s cognitive performance	Freund Clock Drawing Test (CDT) [16], Mini Mental State Examination (MMSE) [17]
Emotional status	Evaluation of the risk for depression	Geriatric Depression Scale—15-item version (GDS-15) [18]
Nutrition	Assessment of patient’s nourishment, weight changes	Mini Nutritional Assessment—Short Form (MNA-SF) [19]
Comorbidities	Assessment of the number of co-existing morbidities and rating accordingly	Charlson Comorbidity Index (CCI) [20]
Polypharmacy	Assessment of the number of medications	Not applicable

## Data Availability

Not applicable.
